# The role of the partner in the support of a pregnant woman’s healthy diet: an explorative qualitative study

**DOI:** 10.1186/s12884-023-06072-9

**Published:** 2023-10-28

**Authors:** Renske M. van Lonkhuijzen, Hanna Rustenhoven, Jeanne H. M. de Vries, Annemarie Wagemakers

**Affiliations:** 1 Department of Social Sciences, Health and Society, Wageningen University & Research, Hollandseweg 1, bode 60, Wageningen, 6706 KN The Netherlands; 2 Department of Agrotechnology and Food Sciences, Human Nutrition & Health, Wageningen University & Research, Stippeneng 4, bode 62, Wageningen, 6708 WE The Netherlands; 3 Dianet, Brennerbaan 130, Utrecht, 3524 BN The Netherlands

**Keywords:** Partner support, Dietary intake, Pregnancy, Empowerment

## Abstract

**Background:**

Active partner involvement during pregnancy is an effective strategy to enhance both maternal and newborn health outcomes. The presence of a supportive partner equips women with a heightened sense of empowerment to deal with the challenges of pregnancy, including maintaining a healthy diet during pregnancy, which is important for the health of both the mother and child. However, little information exists regarding the partner’s role in encouraging a pregnant woman’s healthy dietary choices. This study aimed to explore the perspectives of pregnant women and their partners concerning the partner’s role in promoting a healthy dietary intake during pregnancy.

**Methods:**

Sixteen semi-structured couple interviews were conducted in the Netherlands, involving expecting couples. Based on Berkman’s social networks and support theory, we categorized various forms of support as emotional, instrumental, appraisal, and informational. The interviews were accurately recorded, transcribed verbatim, and analysed using an inductive approach.

**Results:**

In general, pregnant women reported being positive regarding the support they received from their partners. Partners primarily offered instrumental support to pregnant women, such as cooking, grocery shopping, and helping them avoid unsafe foods. Partners provided informational support, mainly about foods considered unsafe during pregnancy. Emotional support was relatively less common. The primary motives for giving support were pregnancy-related symptoms, the importance of the health of the mother and baby, and solidarity with the pregnant woman. Support from the partner was more willingly accepted by pregnant women if the support was perceived as being helpful, showing involvement, and positive. Conversely, partner support was not accepted if it was perceived as judgmental or unwanted.

**Conclusions:**

The majority of pregnant women were satisfied with the support received from their partners, although there are opportunities for a partner to provide more support to improve the dietary intake of pregnant women. To optimise this support, partners are advised to tailor their support to the needs and expectations of pregnant women. Personalizing dietary support can be achieved by couples communicating their dietary wishes and expectations regarding support.

**Supplementary Information:**

The online version contains supplementary material available at 10.1186/s12884-023-06072-9.

## Introduction

 A healthy diet during pregnancy, including a variety of nutrient-dense foods that provide an adequate intake of energy, protein, vitamins, and minerals, while avoiding unsafe foods such as alcoholic beverages, is vital to meet both maternal and foetal needs [1]. A healthy diet is important during pregnancy because it contributes to favourable short- and long-term health outcomes for the mother and child [2–4]. However, overall adherence to dietary guidelines during pregnancy is suboptimal [1, 2]. Inadequate maternal nutrition, characterized by excessive energy intake or micronutrient deficiencies, may lead to several negative health outcomes in the mother, such as gestational diabetes, hypertension, and caesarean delivery [5–7]. Furthermore, an unhealthy maternal diet could also negatively impact the health of the child, including an increased risk of preterm birth, deviation in birth size, and non-communicable diseases in later life such as obesity, diabetes, and cardiovascular diseases [4, 6, 7]. Although most women change their diet by avoiding alcohol, caffeine, and unsafe foods, they tend not to improve their overall diet [6]. Whilst it has been reported that pregnant women should consume a balanced diet according to dietary recommendations, focusing on foods that contain critical nutrients [7], a deeper understanding of the factors that influence their eating behaviour would be valuable.

Social support can serve as an important predictor for both mental and physical health during this critical period [4, 6, 8]. Social support is a fundamental component within the framework of social networks [9, 10]. In pregnancy-related research, defining support is regularly based on the social networks and support theory of Berkman et al. [9, 11, 12]. Berkman et al. [9] theory divides support into emotional, instrumental, appraisal, and informational support, with definitions for each provided in Table 1.

**Table 1:** Definitions of support

*Concept*	*Definition*
Emotional support	Expressions of empathy, love, trust and caring [13]. Emotional support also entails sympathy, understanding and/or esteem or value provided by others [9]
Instrumental support	Help, aid or assistance with tangible needs and services [13]
Appraisal support	Provision of information that is useful for self-evaluation, help in decision making and giving appropriate feedback [9, 13]
Informational support	Provision of advice, suggestions and information in the service of particular needs [9, 13]

Pregnant women who receive substantial social support experience several favourable improvements in their dietary choices [14], including the enhancement of prenatal intake of healthy foods, such as fruits and vegetables [6, 8]. The supporting role of partners is particularly important [8, 15–17]. Firouzan et al. [18] stated that the participation of the father is essential for a healthy pregnancy. The World Health Organization (WHO) indicates that partner involvement, also referred to as male involvement, during pregnancy, childbirth, and the postpartum period is an effective strategy to improve both maternal and newborn health outcomes [1].

However, different interpretations have been given to the term ‘partner involvement’ across the world [15]. In Mozambique, partner involvement was defined as taking care of the family through financial support, participating in decision-making, and expressing affection towards one’s partner. A study in the USA showed that partner involvement was manifested by being present and being emotionally and physically involved, including doing household chores and attentively listening to women’s concerns [15]. Tokhi et al. [17] extended this definition to encourage pregnant women to adopt health-promoting behaviour, such as encouraging a healthy diet and better hygiene practices [17].

While research has been conducted in the field of financial, emotional, and physical support of partners during pregnancy [15–17], studies on partner support for dietary intake in pregnant women are scarce [1, 4, 6–8, 19]. Therefore, the objective of this study was to explore the perspectives of Dutch pregnant women and their partners on the role of the partner in supporting the healthy diet of pregnant women.

## Methods

### Study design and setting

To explore the influence of partner support on the dietary intake of pregnant women, an exploratory qualitative study was performed. Support involves transactions between two people and is therefore an interdependent relationship [16]. In this study, pregnant women and their partners were interviewed using semi-structured couple interviews. Couple interviews offer the unique opportunity of shared storytelling and intra-couple dynamics, add to retrieving rich data [20], and have been suggested as an ‘appropriate method for studying complex shared practices such as making health decisions’ [21].

**Fig. 1:**
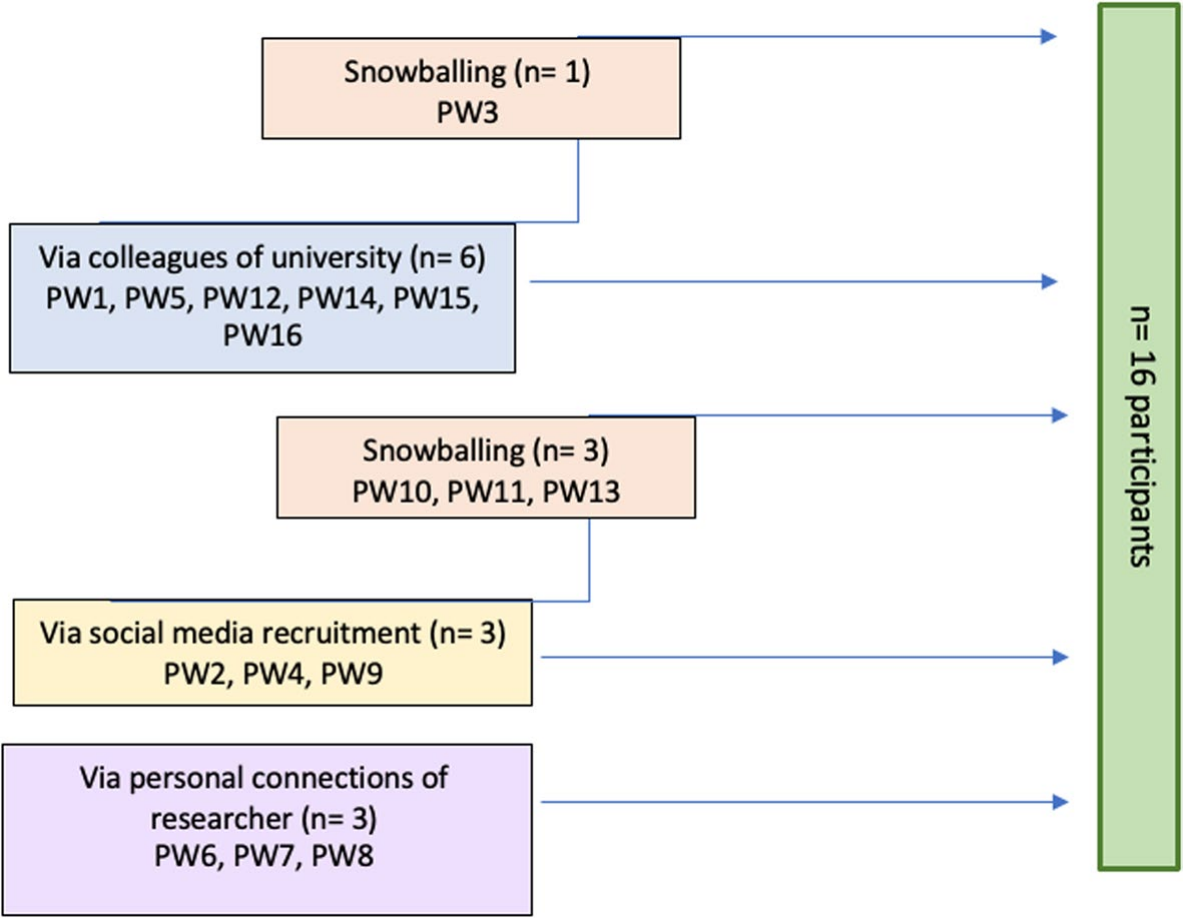
Recruitment and inclusion of pregnant women and their partners

 Recruitment and interviews were conducted between October 2021 and January 2022. In total, 16 couples, and thus 32 participants, were recruited using convenience and snowball sampling (Fig. 1, PW = pregnant woman). The interview invitation was publicly shared online and posted across the researchers’ social networks. The inclusion criteria were that couples spoke and understood Dutch, were in a relationship where they lived in the same household, and the woman was currently pregnant. Upon agreeing to participate, the date and time of the interviews were scheduled. Participants were asked whether they knew of other pregnant couples to participate in the study. Figure 1 illustrates the various ways of how participants were recruited.

### Interviews

The interviews (*N* = 16) lasted between 35 and 63 min, with an average duration of 45 min, and were audio-recorded in Dutch. Most interviews took place online (*n* = 14) due to Covid-19 regulations. In-person (*n* = 2) interviews were conducted in a quiet room of the participants’ choice which ensured confidentiality. All interviews were conducted by interviewer HR with both the pregnant woman and partner present, which made it possible to observe the interaction between the couple and compare perspectives on the subjects during the interview. The characteristics of the couples are listed in Table 2. Data saturation was achieved around interview number 11, but five more interviews were conducted to ensure no additional themes, opinions, or codes occurred in the data [22, 23].

**Table 2:** Descriptive information of couples

Couple	Age (PW, P)	Week of pregnancy at the time of the interview	Pregnant with baby no.	Sex of couple
**1**	35, m.d.^a^	25	1	Male + Female
**2**	22, 23	32	1	Male + Female
**3**	34, 38	25	2	Male + Female
**4**	32, m.d.	20	2	Male + Female
**5**	34, m.d.	38	1	Female + Female
**6**	28, 27	12	1	Male + Female
**7**	25, 28	18	1	Male + Female
**8**	29, 30	37	1	Male + Female
**9**	27, 28	13	2	Male + Female
**10**	m.d., 24	29	1	Male + Female
**11**	32, m.d.	36	1	Male + Female
**12**	27, 31	34	1	Male + Female
**13**	24, 26	36	2	Male + Female
**14**	27, 24	32	1	Male + Female
**15**	23, 24	11	1	Male + Female
**16**	30, 31	12	1	Male + Female

^a^ *m.d. *Missing data

The semi-structured interview guide (Additional file 1) was prepared through a review of the literature and inspired by the research of Super and Wagemakers [24], who learned during co-design meetings that eating during pregnancy is a potentially sensitive topic. Therefore, the interviewer was careful not to judge or comment on the interviewees’ eating behaviour, and to contribute to an open, safe, and supportive environment during the interviews. The interview guide had open-ended questions, ensured that predetermined themes were discussed, and enabled follow-up on new insights during the interview when new interesting themes emerged. During the interviews, the interviewer asked exploratory and clarifying follow-up questions to enable an in-depth understanding of the couples’ points of view.

The interviews were structured into four parts: (1) background information about the couples and their pregnancy, (2) dietary intake before and during pregnancy, (3) perspectives on and acceptance of support, and (4) possibilities to improve support. The questions for parts 1 and 2 were created to gain insight into the background information about the couple and pregnancy. The questions for part 3 were inspired by Greenhill and Vollmer [8], Super and Wagemakers [24], and Berkman et al. [9]. Greenhill and Vollmer [8] focused on the couples’ perceptions of the father’s role regarding a pregnant woman’s nutrition and physical activity behaviour. Super and Wagemakers [24] studied the perspectives of pregnant women on food intake and eating behaviour, experiences of social support, and opportunities for empowerment toward healthy dietary intake. The social networks and support theory of Berkman et al. [9] was used to understand the complexity of social support concerning health and to define four subtypes of social support: emotional, instrumental, appraisal, and informational support. Because women with a supportive partner have been shown to feel empowered to deal with the difficulties of pregnancy [18], the three-step approach of empowerment evaluation was used to formulate the interview questions for part 4 [25]. Research in the area of empowerment in relation to social support and dietary intake is limited [26, 27], but it could support healthy behaviour changes during and after pregnancy. Therefore, identifying opportunities for partners to contribute to the empowerment of pregnant women concerning dietary intake was included in the interviews.

### Data analysis

To ensure anonymity, each participant was assigned a number (couple 1 → PW1 and P1, etc.). The audio recordings were transcribed verbatim and analysed using coding in Atlas.ti, a software for qualitative data analysis. Data were coded iteratively. The interview transcripts were coded inductively by HR. Parts 1, 2, and 3 of the interviews were coded descriptively to easily find the information needed. For example, all quotes about what was eaten daily were coded as Diet_pattern and every complaint experienced during pregnancy was coded as Pregnancy_complaint. This way, an overview was created about pregnancy, diet, and general information such as occupation and age. Part 4 was coded in vivo to remain close to the participants’ perspective. After coding four interviews, an analysis was conducted on the in vivo codes of part four to reveal the underlying structures of experiences or processes [28], and create categories from the data based on these structures. When uncertainties occurred, for example under which codes certain results fit best, this was discussed by all authors to reach a consensus. Coding was discussed and agreed upon by all authors, resulting in a code list (Additional file 2) upon which all 16 interviews were coded. During the process of coding all interviews, only a few new codes emerged. The analysis of the interviews was discussed four times with all authors to reach a consensus.

### Ethical considerations

Ethical approval was granted by the Social Sciences Ethics Committee of Wageningen University and Research on the 20th of October 2021. Participants provided written consent if the interview took place in person, and verbal consent if the interview took place online after they were explained the aim of the study, the procedure of data collection, issues of confidentiality, the voluntary nature of participation, and that they were free to withdraw from participating at any time. Oral permission for audio recording was obtained at the start of each interview.

## Results

The couples were relatively highly educated, seemed happy in their relationship, and felt excited about their pregnancy. The couples were talkative and sympathetic. The sphere of the interviews was open and intimate, which led couples to share personal details such as their health or bad experiences during previous pregnancies. Because the interviews were held with both the pregnant woman and her partner, the interaction between them could be observed, as couples often added to each other’s stories and started asking each other questions. Example of such conversation between two partners:

P9: People often tell stories about men opening a bottle of wine for the pregnant woman to be like: “What about me? I’m pregnant!” Fortunately, we don’t experience that.

PW9: That would not help me or feel as supported either.

P9: No, but if you felt like that, I would not have done that.

PW9: No? Then you would have just continued drinking?

P9: No, then I would not drink wine and stuff. Then I would have just stuck with beer.

PW9 [laughing]: Is that not alcohol as well?

P9: Yes, but I could also open a very nice bottle of white wine, making you feel something like: “I would also have liked that.” However, that is something that I would not do.

When discussing the pregnancy and their complaints, the pregnant women did most of the talking. Some women mentioned not having something to complain about, whereas others experienced complaints such as tiredness, nausea, and vomiting. Partners often indicated that pregnancy was an exciting but difficult time because they empathized with the experience of the pregnant woman. When couples were asked about a rating between 0 and 10 (0 = very unhealthy and 10 = very healthy), they would assign to the healthiness of their diet: they graded this at about 7 out of 10 on average, ranging from 5 to 8.5. Only one couple scored their diet as low as a 5/10.

P13: With all snacks I eat… I think I am at a 5.

PW13: I think I also score 5 because I keep eating all day because I am hungry all the time.

P13: Yes, you will then grab a cookie or some candy…

PW13: Yes, to stop nausea, I just need to eat.

Dietary changes since pregnancy differed between couples. Almost half of the couples mentioned being more occupied with being healthy during the pregnancy than before. Some pregnant women consume more vegetables and fruits, while others consume more unhealthy snacks, often caused by pregnancy cravings. All pregnant women changed their diet by withholding unsafe foods such as raw meat, soft cheese, and alcohol.

“I noticed that when I found out I was pregnant, a switch flipped [to eat healthier].” (PW16)

When discussing support, almost all couples agreed with one another regarding their provided dietary support. Sometimes it was difficult for couples to come up with examples of dietary support, but most of them would add to each other’s previously made suggestions. For some couples, the pregnant women primarily supported their partner with a healthier diet, whereas in other couples the partner mostly supported the pregnant woman.

### Partner support

When the couples were asked what came to mind when talking about support during pregnancy, most of them started talking about non-dietary support. Partners were more involved in doing household chores since pregnancy by vacuuming, doing the laundry, or loading and unloading the dishwasher. In addition, partners expressed more empathy and encouragement towards pregnant women. These were either related to pregnancy complaints, such as being tired or feeling sick, or being interested in pregnancy itself.

#### Instrumental dietary support

The majority of pregnant women received a lot of instrumental dietary support, including the partner cooking, doing groceries, and withholding from foods that are unsafe during pregnancy. During pregnancy, partners cooked more often than before. This included preparing dinner together with the pregnant women or doing it more often by themselves. Partners also took more care of the pregnant women during the day, for example by preparing fruit, ensuring that she drank enough fluids, or bringing a cup of tea. Partners also went grocery shopping more during pregnancy. Some partners mentioned accompanying the pregnant women to the store to carry the heavy bags, whereas others said they took on the entire task of grocery shopping.

“You did a lot of cooking, you made sure that the groceries were done, that there was enough food in the house, and that there was plenty of vegetables and fruit.” (PW5).

Pregnancy-related complaints among pregnant women have caused their partners to increase instrumental dietary support (e.g., cooking or groceries). Complaints such as tiredness or nausea made it more difficult for pregnant women to cook or do groceries, so the partners felt that they needed to take on these tasks. Being available helped the partners provide support. Some partners, for example, mentioned that the COVID-19 regulations were beneficial for providing support since they were more at home to help around the house.

“You see, on days that she is really tired or that we did something, and we get home and she is out of energy, then I often cook.” (P12).

“There was also a phase when you couldn’t handle standing in the smell of cooking so during that phase I did it more often.” (P13).

All pregnant women withheld unsafe foods during their pregnancies. 15 of the 16 pregnant women used the Zwangerhap app of The Netherlands Nutrition Centre to determine what to eat during pregnancy. Partners supported pregnant women by also withholding themselves from unsafe foods and felt like it would not be solidary to eat them in the presence of pregnant women. Ten partners mentioned drinking no or significantly less alcohol in the presence of the pregnant women or withholding from eating unsafe foods, such as raw meats and unpasteurized cheeses because of pregnancy. Partners felt that it would make it easier for pregnant women to avoid certain foods if they withheld themselves as well, and felt that it was not fun to eat or drink these foods alone.

“I think it is the biggest support that you do not eat everything that I cannot eat right in front of me, for example ordering sushi while I cannot have that.” (PW9).

“He says: I am not going to drink wine alone. You are sitting next to me, and I am drinking a glass of red wine by myself. That’s just not as nice.” (PW14).

“I understand that people do not want the smell of salmon in their nose when they cannot eat it” (P5).

#### Appraisal dietary support

Appraisal support refers to providing support for self-evaluation, helping in decision making, and giving appropriate feedback. Over half of the partners gave appraisal dietary support to increase the pregnant women’s consciousness of their dietary intake. Partners made comments about the pregnant women eating unhealthy food or steered them to eat healthier foods. Some partners were concerned about the amount of food consumed in a day and made comments regarding this.

“I once said that I really wanted to go to a snack bar and then he said; we are not going to do that, you will be grateful later.” (PW3).

“I can’t drink too much coffee. Only two cups a day, and sometimes when I say I would like another one, he says: you know you don’t need to drink more than two cups of coffee per day” (PW14).

In addition to increasing awareness of dietary intake, five partners specifically provided appraisal support for unsafe foods. For example, while doing groceries, partners asked which foods the pregnant women were allowed to eat and thus could be taken home, or while preparing meals which foods could be eaten. Some partners also commented on which foods should be eaten by the pregnant women or made suggestions about what to eat while being nauseous.

“He always asks something like: I am making fresh mint tea; can you have that?” (PW11).

“I try to say to her: you need to eat something light, like yoghurt, which includes a lot of protein so that you gain some strength back” (P15).

The importance of a healthy diet and avoiding unsafe foods were reasons for the partners to provide appraisal support for the health of both the mother and the baby.

“Raw meat is just something we are not going to do […] why would you risk that?” (P4).

#### Informational dietary support

Most informational support provided by the partner concerned foods that were unsafe during pregnancy. Partners informed themselves about these food products, discussed them with the pregnant women, and checked on food labels what could and could not be eaten by pregnant women. Some partners downloaded pregnancy- and dietary-related apps on their phones to inform themselves.

PW13: He makes sure that I do not eat something that I cannot have because of my pregnancy.

P13: Yes, with salmon, steak, or something like that.

PW13: Yes, like can you have that? So, you do not look at what is healthy, but you do look at what can be harmful to the baby.

In one interview, informational support regarding additional nutrients during certain period of pregnancy was provided.

“The app said, for example, that in this week the bones are being developed, so calcium is important. So, then I say: you need a bit more yoghurt.” (P5).

Informational support was often provided because the partner felt nervous about the pregnancy. Many of the partners expecting their first child felt uncertain in the first few weeks of pregnancy, since it was a new experience for them. Some partners were worried about the pregnant woman feeling sick. Information was sought to feel more confident and know what to eat during pregnancy. Similar to appraisal support, the health of the mother and baby underlies the reasons for providing this type of support.

“Especially in the beginning I was quite nervous about how it would all go up until 12 weeks, so then I asked many times whether she feels good and whether it wasn’t too heavy carrying certain things and all those kinds of things.” (P5).

#### Emotional dietary support

Emotional dietary support was mentioned in four interviews and was thereby the least mentioned type of dietary support during pregnancy: partners supported pregnant women by expressing care and empathy regarding dietary difficulties.

“At moments that it gets difficult, I try to empathize even though it is difficult since you do not feel it yourself. And also, to talk about it helps a lot if she can just share her story.” (P15).

#### No support received by pregnant women

In some situations, dietary support was not received by the pregnant woman and was not provided by the partner. Examples of such situations were that the partners did not withhold themselves from eating pregnancy-unsafe foods, were not involved in the dietary intake of the pregnant women, or did not take on tasks such as doing groceries. The main reason for not providing dietary support was that it was not needed or appreciated by the pregnant woman, for example, because the pregnant woman was well aware of unsafe foods, was already eating healthy, or was not experiencing difficulties. Some partners did not feel comfortable telling the pregnant women what (not) to eat. Not consciously being occupied with pregnant women’s dietary intake is also a reason why partners did not provide dietary support. Some partners expressed a lack of knowledge about what is healthy and what are the important nutrients during pregnancy. Lastly, pregnancy complaints, such as nausea, made it difficult for partners to provide dietary support because they did not know which foods to advise during nauseous periods or felt that pregnant women were less approachable. Partners also expressed that pregnant women should be able to enjoy (unhealthy) food during pregnancy without receiving comments about it.

### Support acceptance

In a minority of cases, the support provided by partners was not accepted by pregnant women. For all four types of support, there were examples of pregnant women accepting support and pregnant women not accepting support. For example, regarding instrumental dietary support, some pregnant women accepted that their partner did the groceries, while others did not want their partner to do so. Appraisal support was accepted by some pregnant women because they felt that it showed involvement. However, others did not like appraisal support as it felt like unwanted interference.

“He sometimes bought me brown bread and then I got irritated because I don’t want brown bread – I want white bread! […] The more people pay attention to things I should not do, the more I feel the urge to do them. [.] So I think; what are you meddling? As if I’m some dumbass who does not know how to take care of herself.” (PW6)

#### Reasons for acceptance of support by the pregnant woman

Appreciation was the main reason for accepting support from their partners. In eight interviews, pregnant women appreciated getting support, from their partners or relatives. Multiple reasons can be identified as to why the given support has been accepted. One example is that support was accepted because it was helpful for pregnant women to take away some of the burdens of household chores. Another reason for support acceptance was that the support showed involvement and was perceived as being caring.

P8: I try to pay attention to what I eat. Because if I would eat crisps, or when I would get McDonald’s, I know that she would want it too. It would not be fair to eat it alone, so I think that I would pull her into that unhealthy food if I did that.

PW8: Yes, I am happy about that because I cannot guarantee that I am strong enough to resist that.

“I think that’s so sweet, that he is so involved in my health in that way” (P6).

The origin of support is relevant to whether it is accepted. Pregnant women more readily accept support when given by an expert, such as a midwife or a dietician. Besides experts, support is also more willingly accepted by someone who is also pregnant or has been pregnant before. Two participants, for example, more readily accepted support from their mothers than from their partner.

I: What if you received a dietary tip from a midwife, would that be different?

PW8: Yes, I think it is different. You can tell that they received training on how to transmit information. […] With midwives, it is their occupation, they have studied for it, and they have experience. I accept this information more willingly.

In addition to the importance of the person who provides support, the way support is communicated is important. For example, support should not be communicated with a know-it-all attitude or coercively but rather as advice to be accepted. The provided support should be clearly explained and come from a reliable source. Some pregnant women would like support to be given directly, but others prefer it more subtly.

“I always prefer that you just communicate directly to me” (PW12).

“I do need to communicate it subtle otherwise it will come across as an attack” (P14).

Support was more likely to be accepted if it was requested, not repeated constantly, and when provided at the beginning of the pregnancy rather than later or during a second pregnancy. This is because couples have gathered more information and experience later in pregnancy, reducing the need for support at later time points.

P3: Yes, I think support leads to healthier choices.

PW3: I think so too. I have quite some knowledge, so I do not need it as much. However, I do think that if you would have asked this question during my first pregnancy, I think I would have answered differently. I searched for a lot of information about the types of fish, etc. I was like a walking Google during my first pregnancy.

Finally, the content of the support was important. Pregnant women would receive positive rather than negative feedback, and would therefore rather receive support about what is healthy than what is unhealthy. However, some support regarding what is unhealthy or what can be harmful to the baby is appreciated. Other dietary support provided by the partner was only accepted by the pregnant woman if they felt that it was relevant. For example, if the partner provided appraisal support regarding snack behaviour (e.g., eating too many biscuits), it was only accepted if the pregnant woman agreed that her snack behaviour was problematic. In addition, if the partner provides this kind of support, the partner should comply with the support they provide. Support regarding unhealthy snack behaviour will only be accepted by pregnant women if the partner has healthy snack behaviour.

I: So when he tells you ‘This is healthy and I know you would like it’, that would be nice and you will take the support into account, but if he says ‘you should not eat this’, it would be harder to accept?

PW15: It depends, if I would eat cookies all day, and he would say: maybe that is not clever to do, it would be different. However, if I just eat one cookie occasionally, I would just enjoy eating it.

#### Reasons for non-acceptance of support by pregnant women

In the few cases of non-acceptance of support by pregnant women, some reasons were mentioned by both the pregnant women and their partners. Some characteristic traits of pregnant women have been mentioned as reasons for non-acceptance, such as stubbornness, recalcitrance, and being bad at receiving support. Partners find it more difficult to give support to pregnant women when they know it will not be appreciated by them, due to their character or previous experiences of failed attempts to provide support.

“I have a hard time letting go. I often think I can do it better. […] Even if I should stop and lie down, I am stubborn. I usually only sit down if I am done doing everything around the house.” (PW3).

“If I am being honest, I am really bad at receiving criticism or unwanted advice.” (PW6).

Pregnant women may feel that their partners do not have the authority to decide what they can eat. Most pregnant women said they were able to decide for themselves what is good or not good to eat and indicated that they already had a healthy diet before pregnancy, so the involvement of the partner was not appreciated. Support was also not accepted because of acute urges such as pregnancy cravings. If pregnant women want to eat a certain thing, it does not matter how support is communicated: it would not be accepted by pregnant women anyway.

“I’m like, it is my life and my body, and I can decide what I eat or drink” (PW6).

Dietary support can also have negative outcomes. When partners, for example, gave appraisal support for improving snack behaviour, pregnant women felt more urged to eat snacks.

I: If your partner would say something like ‘Maybe you should not drink the glass of chocolate milk.’ What would you think?

PW14: I would then drink an extra glass of chocolate milk.

Support was not accepted when it felt as judgment or unwanted interference. Some pregnant women felt judged for their choices regarding unsafe foods. Trustworthiness and unclear advice were other reasons for not accepting support. Most couples indicated that it is important to follow dietary guidelines, and support is less likely to be accepted when it is not in line with these guidelines. In rare cases of someone providing advice that conflicts with these guidelines, such as ‘one sip of alcohol will not harm the baby,’ this was often not accepted and appreciated.

P5: My mother tried to give you alcohol, which was very weird. I was really confused because she is not a drinker at all. She usually feels disgusted with alcohol consumption.

PW5: But she did so because your dad made wine of its own.

P5: Yes, my dad made wine, but then I said: ‘You will not feed alcohol to my baby, right Mom?’

Pregnant women mentioned that they should be able to follow the provided support for it to be accepted. One participant mentioned that she did not listen to the appraisal support of their partner regarding the amount of food she eats because she was unable able to eat large amounts of food anyway because of her pregnancy. Support is also not accepted when it is about eating food products that they do not like. For example, if a partner advises a pregnant woman to eat more vegetables, even if she does not like vegetables, the support would not be accepted.

“At the beginning when you are pregnant and everything is new, you get a lot of recommendations. In addition to supplements and vitamins. After a while, I stopped taking them because I never took them in my life, and I found it weird to do it now. Besides, those pills were gross.” (PW1).

### Satisfaction with dietary support

Of the 16 couples, 11 were satisfied with the dietary support between the two; in two couples the pregnant woman and her partner were both not satisfied, and in three couples the pregnant women were satisfied but the partner was not satisfied with the support they provided. PW2 mentioned that they stimulated each other into an unhealthy diet at times. When asked whether her partner could support her in eating healthier, she said the following:

“We are quickly inclined to pull each other into an unhealthy diet while in most cases we have plenty of time to cook and eat a healthy meal. […] When I think about dinner, I would find it nice if he would stop me more often from eating unhealthy food.” (PW2).

Between couple 13, the following conversation took place when talking about the dietary support provided by the partner:

P13: Our son eats fruit every day. I am more occupied with him eating fruit than you eating healthy

PW13: Yes, I think you forget at times that I need to live healthily because I am pregnant.

P13: Yes, our son doesn’t eat very well so I find it important that he eats fruit

PW13: And then you forget about me.

P13: Yes, I forget about you a bit, even though I am already busy preparing fruit, so I could also make you some. I may fall a bit short there.

PW8 stated that she was happy with the support she had received. However, while discussing support during the interview, she realized that she would have liked more informational support from her partner.

“We could have delved a little bit more in the information about what nutrients are good during the development of the baby in the womb.” (PW8).

When partners were asked whether they were satisfied with the support they provided, five made suggestions for improvement. Some partners mentioned that they did not consciously think about the dietary patterns of the pregnant woman before, and that they could be more interested in this. Others mentioned that they could improve the provision of healthy meals and snacks. P15 was unsure about providing support because he did not know what to advise and what was healthy during pregnancy, so he mentioned that he could improve on this matter.

“I am partly happy with the support I provide. It could be improved, but I am often unsure about what the best choice is. I could give a lot of healthy suggestions, but I don’t know if that is also healthy during pregnancy or in combination with nausea.” (P15).

“I encourage her to keep eating well, but sometimes in a bit of a snappy way. […] I could let go of that snapping a bit more… But at least I try to support her.” (P16).

## Discussion

This study explored the perspectives of pregnant women and their partners on the role of the partner in the support of the pregnant women’s dietary intake. To our knowledge, this is the first study to conduct couple interviews to explore the role of partners in supporting pregnant women with a healthy diet.

Partners provided all four subtypes of social support: emotional, instrumental, appraisal, and informational. The most provided type of support was instrumental dietary support, encompassing cooking, doing groceries, and withholding unsafe foods such as raw meats and alcohol. This is in line with findings about dietary changes of first-time parents in Belgium, as Versele, et al. [4, 19] found that social support by providing a healthy meal or doing groceries could help provide a healthy home food environment during pregnancy. When giving appraisal dietary support, partners in this research encouraged pregnant women to increase their awareness of a healthy diet and to stimulate their self-reflection about the healthiness of their diet, the amount of food consumed, or the use of unsafe foods during pregnancy. Informational dietary support is mostly provided for unsafe food. This was also found by Super and Wagemakers [24], who explained that partner support could offer nutritional and practical tips for healthy eating during pregnancy.

Most of the pregnant women were satisfied with the dietary support they received from their partners, but no conclusions could be drawn on what type of support was better accepted or appreciated by pregnant women. Pregnant women differed in the ways they liked their partners to communicate support, such as a more subtle versus a more direct approach. In this study, pregnant women preferred the support they asked for, instead of unwanted interference with their diet. However, in the literature, having to ask for support may not be in line with the norms of intimate ties, as having to ask a partner for support may be viewed as the partner being uncaring or inattentive to one’s needs [16]. Heaney and Israel [10, 13] stated that more intimate ties, such as partners, are better at providing emotional support and more distant ties are better at providing informational support. Interestingly, there was barely any mention of emotional dietary support provided by partners when discussing dietary support in this research. However, the Dutch couples in this research mentioned emotional support often when discussing support in general. A possible explanation for this could be that it is common for Dutch men to view emotional support as a normal part of being in a relationship instead of seeing it as additional support. Providing emotional dietary support might be self-evident, and less worth mentioning. Related to this, partner support may fit multiple types of support and could overlap. For example, buying healthy food can be viewed as both informational and instrumental.

Partners of pregnant women can and do play a role in the change towards healthy behaviour by providing support. In line with previous research on partner involvement during pregnancy by Rhodes et al. [29], the couples in this study appreciated partner support to form a team to achieve dietary goals. This study provides opportunities to improve the dietary support for a healthy diet for pregnant women. Based on the different perspectives on the support of the expecting couples and the differences in support acceptance, tailored support is needed to support and empower pregnant women towards a healthy diet. According to Rini et al. [16], support can be of higher quality when it meets the receiver’s needs, as even well-intended support can backfire if the wants and needs of the support receiver and support giver do not correspond. Super and Wagemakers [24] stressed the importance of open dialogue with pregnant women to identify meaningful issues regarding healthy eating habits. However, many couples did not discuss matters such as dietary patterns or dietary support before the interview. Therefore, couples are advised to have a conversation about dietary intake and support, where pregnant women can express what their ideal dietary intake would look like and what is needed to achieve that. The couple can then discuss what dietary support is currently provided by the partner, whether that support is accepted, and how support should be communicated. These results suggest that support should be communicated in an advisory manner rather than coercively or know-it-all. The quality of support diminishes when it is received negatively on the efficacy or worthiness of the support receiver, in line with the research of Rini et al. [16]. As found in this study and as stated by the WHO [1], nutritional support should be communicated positively. These findings show that partners should focus on what is healthy for the pregnant woman and what is going well regarding their diet.

Women empowerment is commonly believed to improve health, including during pregnancy and childbirth [30, 31]. If a population is empowered to be involved in health promotion regarding nutrition, dietary improvements are more likely to be sustainable and effective than top-down educational strategies [32]. Consequently, not only should women feel empowered, but also their partners should feel empowered to provide dietary support for pregnant women. Further research should investigate strategies that support partners in discussing dietary intake with pregnant women. Interventions that focus on healthy nutrition communication to improve empowerment, such as Power 4 a Healthy Pregnancy, could be promising to help facilitate these discussions between couples [33].

A study on the influence of the partner on the dietary intake of pregnant women, including couple interviewing, has never been conducted before. A strength of this study lies in the execution of the interviews with couples, which enriched the data by them adding to each other’s stories and asking each other questions about the subjects discussed [34], which in itself is an example of how the interviews contributed to the empowerment of the couple. Bjørnholt and Farstad [20] found in their research on couple interviewing that it can open up new and interesting knowledge and can place the researcher in an observing role as the partners have discussions among themselves, which also occurred in this research. Because couples challenge and correct each other’s narratives, the data tends to be more realistic and less idealised [20]. However, couple interviews might prevent the collection of sensitive information that respondents do not want their partner to know [35]. Most interviews were carried out online due to COVID-19 regulations, which is positive because of health concerns and because it allows for interviewees and interviewer flexibility. However, limitations of online interviews could be technological problems, difficulty in ensuring that participants feel at ease, or a feeling of distance which could reduce their commitment to the process [36]. Limitations of the couple and online interviewing were not encountered in this study. The study sample originated from various parts of the Netherlands; however, although the level of education was not systematically collected, the interview results indicate a relatively highly educated sample. As educational level is related to diet quality, this could have resulted in a study population with better diet quality and food literacy than the average Dutch population. Gestations of pregnancy varied widely in the study sample, which is helpful for gaining a better understanding of the need for support throughout pregnancy. As this explorative, qualitative study did not aim to generate a generalisable result, but rather to gain an in-depth understanding of the situation, future research on partner dietary support among couples of diverse (educational) backgrounds could add further important insights to this study. Additionally, the possibilities for support in the various gestations of pregnancy can be explored further in future research.

## Conclusion

Nutrition during pregnancy is important for both the mother and baby. An unhealthy diet can lead to negative health outcomes such as gestational diabetes and hypertension. This study explored the perspectives of pregnant women and their partners on the role of the partner in supporting pregnant women’s dietary intake. Partners in this study were mostly involved in the pregnancy and diet of the pregnant women by providing instrumental support by doing groceries, cooking, and withholding from foods that are unsafe during pregnancy. Appraisal and informational dietary support were also provided by commenting on a healthy diet and foods that can be unsafe during pregnancy. Dietary support was provided by partners to be solidary to the pregnant woman because of pregnancy complaints or the importance of the health of the mother and baby. Support was received and appreciated differently among pregnant women. Therefore, the suggestion for partners to make dietary support more effective is to personalize dietary support according to the needs and expectations of the pregnant woman. Communicating these needs as a couple has the potential to improve the empowerment of both pregnant woman and their partners.

### Supplementary Information

**Additional file 1.** Interview guide.

**Additional file 2.** Code list guide.

## Data Availability

The data will not be made publicly available to protect the participant’s identity. Data are available from the authors upon reasonable request and subject to approval from the ethics committee. Data requests can be sent to renske.vanlonkhuijzen@wur.nl.
