# Supplementary material for: The role of the partner in the support of a pregnant woman’s healthy diet: an explorative qualitative study
Source: BMC Pregnancy Childbirth. 2023 Oct 28;23:760. doi: 10.1186/s12884-023-06072-9 (PMC10612286; doi:10.1186/s12884-023-06072-9)
Supplement: Supplementary file 1 — Additional file 1. Interview guide. [file 12884_2023_6072_MOESM1_ESM.docx]

# Additional file 1

Interview guide: The role of the partner in the support of a pregnant woman’s healthy diet: an explorative qualitative study

| **Research topic** | **Questions/topics** | **Theme/purpose** |
| --- | --- | --- |
| Background information about the couple and their pregnancy | Age  Family situation  Education  Work  How long have you been together?  How long have you been living together?  Number of weeks pregnant  Previous pregnancies  How is the pregnancy going so far? *– What's going well? What have been difficult moments? Do you experience physical or mental complaints?*  Have you ever been referred to a dietitian during this or a previous pregnancy? | - Gain insight into the context, relationship, and living situation of the couple  - Gain insight into the context and pregnancy of the couple |
| Diet of the pregnant woman and partner and change during pregnancy | Are you focused on healthy food during pregnancy?  What do you think of when I talk about healthy food?  What do both of your diets look like? Can you describe what you generally eat on an average day?  Do you have a different diet now that you're pregnant?  What rating between 0 and 10 (0 = very unhealthy and 10 = very healthy) would you assign your diet if you were not pregnant? *– Why?*  Do you find it difficult to eat healthy during your pregnancy?  Did you receive nutritional advice during this pregnancy? | - Gain insight into images and ideas about healthy food.  - Insight into the dietary pattern for the pregnancy of both pregnant woman and partner  - Understanding the change in diet since pregnancy |
| Social support | When I talk about social support, what do you think of?  Could you tell us a bit about what kind of support you experience from your environment now that you are pregnant? *– In which areas do you experience support? How often? Do you accept this? What do you do with the received support? Do you experience the support as positive or negative? Why?*  Do you receive support regarding nutrition? *– How often? Do you accept this? Do you do something with the support? Do you adjust behavior based on support? Do you experience this support as positive or negative? Why?*  What do you experience in terms of support from your partner? *– In which areas? How often? Do you accept this support? Do you do something with this support? Do you adjust behavior based on the support? Do you experience this support as positive or negative? Why?*  Do you think you're more likely to take support from your partner or someone else?  To partner: Do you feel that you are supporting your pregnant partner when it comes to nutrition? *– How? How often? Do you feel that your support is appreciated and accepted?* | - Perspective on the definition of social support  - Insight into what types of support are received by a pregnant woman (emotional, instrumental, appraisal, or information)  - Gain insight into received (nutrition) support from partner (step 2 of empowerment approach)  - Insight into support reception intimate ties vs. distant ties  - Insight into the perspective of the partner in terms of giving and receiving support |
| Possibilities to improve support | Would you like to receive more or less support from your partner regarding nutrition? *– What does that look like? What kind of support would you like to receive? What makes support from your partner pleasant for you? What would help you to accept the support from your partner?*  To partner: Could you improve the support you provide? *– Why? How?*  Do you think getting support leads to healthier food choices? *– Why?* | - Gain insight into the perspectives of the pregnant woman and partner on how support can be improved (plan for the future; step 3 empowerment approach)  - gain insight into how the social environment can be navigated (Super and Wagemakers) |
