# Supplementary material for: The role of the partner in the support of a pregnant woman’s healthy diet: an explorative qualitative study
Source: BMC Pregnancy Childbirth. 2023 Oct 28;23:760. doi: 10.1186/s12884-023-06072-9 (PMC10612286; doi:10.1186/s12884-023-06072-9)
Supplement: Supplementary file 2 — Additional file 2. Code list guide. [file 12884_2023_6072_MOESM2_ESM.docx]

# Additional file 2

Code list: The role of the partner in the support of a pregnant woman’s healthy diet: an explorative qualitative study

**General information**

- Age
- Occupation
- Residence
- Study

**Relation**

- Relation_livingtogether
- Relation_status
- Relationship_yearsofbeingtogether

**Pregnancy**

- Pregnancy_complaint
- Pregnancy_differencebetweenpregnancies
- Pregnancy_experienceofpreviouspregnancy
- Pregnancy_firstchild
- Pregnancy_secondchild
- Pregnancy_specialmoment
- Pregnancy_status
- Pregancy_weeks

**Diet**

- Diet_beforepregnancy
- Diet_changesincepregnancy
- Diet_confusinginformation
- Diet_difficulties
- Diet_grade
- Diet_ideasabouthealth
- Diet_occupiedwithahealthydiet
- Diet_pattern
- Diet_pregnancycraving

**Support between couple**

- P_givesdietarysupport_informational
- P_givesdietarysupport_instrumental
- P_givesdietarysupport_emotional
- P_givesdietarysupport_appraisal
- P_givesnondietarysupport
- PW_givesdietarysupport
- PW_givesnondietarysupport
- Reasons_givingsupport

**Other support**

- Relatives_supportcouple
- Professionals_supportcouple
- Support_unhealthyeating

**Amount of support**

- Support_notreceived
- Support_moreneeded
- Support_extraseeked

**Reactions to support**

- Support_accepted
- Support_dependanceofacceptance
- Reasons_supportaccepted
- Support_notaccepted
- Reasons_supportnotaccepted
- Support_notneededforPW
- Support_notneededforcouple

**Perspective on support**

- Perspective_PWcontentwithsupport
- Perspective_Pcontentwithsupport
- Perspective_couplecontentwithsupport
- Perspective_PWwantsmoresupportfromP
- Perspective_Pnotcontentwithsupporttheygive
- Perspective_supportleadstobetterhealth
- Reason_perspectivesupportleadstobetterhealth

**Codes later added**

- Reason_support not given
- Reason_support not received
- Reason_moresupportneeded
- Reason_supportnotneeded
- PW_notcontentwithsupport
